# Use of a Social Media Platform for Undergraduate Medical Writing: Experience From a Developing Country

**DOI:** 10.7759/cureus.87971

**Published:** 2025-07-15

**Authors:** Sayuri Serasinghe, Umanga Geekiyanage, Buddhima Premaratne, Rohan Siriwardana

**Affiliations:** 1 Surgery, Colombo North Centre for Liver Diseases, Faculty of Medicine, University of Kelaniya, Colombo, LKA

**Keywords:** facebook, medical writing, scholarly communication, social media, undergraduate medical education

## Abstract

Introduction

While scientific writing is an important skill undergraduates need to develop, many find it to be challenging in the early parts of their journey, as opportunities to improve and publish their academic writing are scarce. Although many scientific journals use social media platforms to increase their accessibility, their use in undergraduate writing is not documented.

Methods

The medical student journal “The Apprentice” and its Facebook platform were established in 2020 by a volunteer group of medical students representing all academic years. The Facebook journal publishes case reports, scientific papers, posts of medical interest, and posts containing matters of medical education and perspectives. Written work submitted to the journal is reviewed by the editorial board, which consists of a volunteer group of students. The final review is done by the volunteer staff advisors. Subsequently, work is published on the Facebook platform. The three-year progress of the journal was evaluated by looking at total likes, interactions, and reaches of the published posts.

Results

Over a period of three years, there were 3,344 followers and 3,269 likes. There were 202 posts, with a mean of 333 reaches per post and 44 mean engagements. Most of the journal page followers were in the age range of 25-34 years, which coincides with the majority of undergraduates in Sri Lankan medical schools. Additionally, they were geographically centered around the three main universities in the country. The highest reach and engagement were seen with the case reports (reach: 995) and original articles (reach: 1980) posted on the page.

Conclusions

This study demonstrates the ability to use a social media-based student journal to bridge the gap between informal learning and formal academic publishing, especially in a resource-poor setting. “The Apprentice” Facebook journal became popular among medical undergraduates in a short time.

## Introduction

The ability to think and write critically directly reflects an academic’s ability to share their work and innovations with other academics and researchers [1]. Developing this skill, therefore, becomes an essential part of an undergraduate’s learning curve, and many find it challenging in the early parts of their journey [2]. A method commonly utilized in improving the thinking process is engaging in reading academic texts and reviewing them in group settings, such as in the traditional “journal clubs” [2]. As academic writing, especially intended for publication, is an anxiety-inducing adventure for many, being exposed to writing and engaging in research work since the early days of undergraduate training is associated with academic success and persisting with research in later life [3,4]. However, in many countries, research training during undergraduate days is still underdeveloped [5]. The opportunity and access to publish in mainstream medical or scientific journals are limited to medical students, which may discourage them from disseminating their research findings and considering a future in academics [3]. To tackle this, several medical student journals (MSJ) have been established worldwide with the objective of providing a student-friendly environment for undergraduates to improve their writing and familiarize themselves with the publication process [6].

In recent years, the use of social media in medical education has become popular as it holds a remarkable potential to help enhance the learning process [7]. Social media promote active learning with their stimulating content and interaction and are known to be flexible, which leads to customization of the learning experience to fit the learner’s needs [8]. Facebook is one of the most widely used and influential social media platforms worldwide in medical education [8,9]. Many top scientific journals and academic institutions maintain their social media platforms to popularize their research publications among a wider audience, highlighting the strength of social media. Social media, with its popularity among young adults and its liberty to access and evolve [9], makes it an exciting place to create a platform for academic writing for undergraduates. Although the use of social media broadly in medical education has been assessed before, the significant gap in understanding how social media platforms can be used to improve undergraduates’ engagement in academic writing and reading has not been assessed previously, to the best of the authors’ knowledge. This study was designed to address this gap by evaluating Sri Lanka’s first MSJ’s social media platform, specifically analyzing the content preferences, user engagement patterns, and demographic reach to assess the feasibility of using social media platforms to enhance undergraduate engagement in academic writing.

The MSJ “The Apprentice” and its Facebook page were established in November 2020 (https://www.facebook.com/apprenticejournal), with the primary aim of enhancing academic writing skills and publication skills in undergraduates. A volunteer group of students in the Faculty of Medicine, University of Kelaniya, Sri Lanka, primarily manages the Facebook page. The organizational structure comprises an editor-in-chief representing the final year of medical school, subeditors representing each batch of medical students, and volunteer representatives from various batches. Volunteer academic staff members in the departments of surgery, medicine, and pediatrics contribute as mentors.

Medical students are given the opportunity to submit posts they have authored in different areas through their batch representatives. They are encouraged to adhere to the author guidelines of the journal, which were established by the editorial board with the guidance of the advisors. Posts on medical education, case reports, and research papers, once created, are reviewed by student editors and later by staff advisors before publication on the Facebook page. Once submitted, the students are given feedback and assistance in improving their articles till publishing. They are also encouraged to request any assistance in writing they require, which is provided by the editors and staff advisors. This study evaluated the three-year outcomes of this initiative.

## Materials and methods

For this study, the Facebook activities were arbitrarily categorized as described in Table 1. We analyzed the page activities from November 1, 2020, to April 30, 2024 (three years and five months). Facebook page analytics were extracted in May 2024. Miscellaneous categories of posts, which contained notices and advertisements, were not considered for this study.

**Table 1:** Categorization of Facebook posts

Category	Description
Brief posts of medical interest	Posts related to medicine but do not contain any material related to medical education
Brief posts on medical education	Posts containing factual matters related to medical education
Intermedical faculty activities	Posts related to intermedical faculty events conducted by the forum
Case reports	Medical case reports solely published on the Facebook page
Perspectives	Individual perspectives of student-related areas
Publications	Research papers by medical students based on their undergraduate projects
Miscellaneous activities	Consists of notices for academic sessions and other announcements

Basic statistics included overall page statistics, likes, and the demographics of viewers. Demographic data were collected from Facebook’s native analytics platform - Facebook Insights, which provides anonymized demographic information regarding page followers. It contains data automatically collected and summarized by Facebook from the Facebook page. Further, we analyzed each category of posts for total engagements and post reach.

We analyzed the performance of a post using the “reach” and “engagement”. According to Facebook Help Center, “reach” is defined as “the number of people who saw the post at least once” [10]. “Engagement” is defined as “the number of times members engaged with the post through reactions, comments, shares, photo or video views, and clicks” [11].

For analytical purposes, for each category, we took the mean reach as the total reach divided by the number of posts and the mean engagement as the total engagement divided by the number of posts. We calculated the engagement rate for a post as engagement of a post/reach of a post × 100 and calculated the mean for each category.

Statistical analysis was limited to descriptive statistics, including means, totals, and percentages, as this represents the first evaluation of a social media-based MSJ. No inferential statistics were performed, as this was a descriptive analysis of social media engagement metrics over time. The limitations of this descriptive approach include the inability to establish causal relationships of statistical significance between variables.

As this study was done using data available online and does not directly involve individual subjects, no specific ethical considerations were present.

## Results

**Figure 1:**
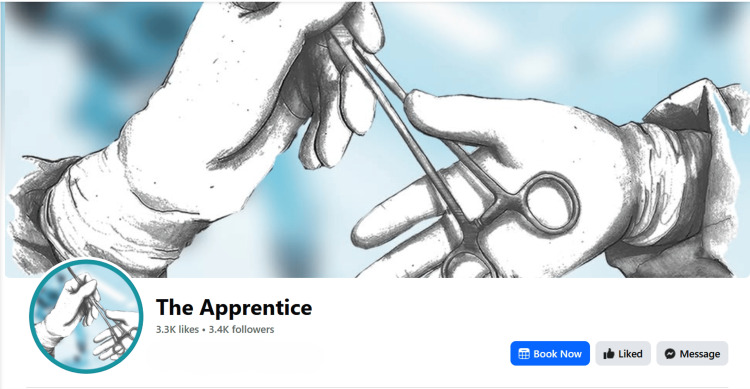
Screenshot of The Apprentice Facebook page

Figure 1 shows the homepage of The Apprentice Facebook page as it appeared during the study period. Over the period of three years, The Apprentice’s Facebook page had 3,344 followers from November 2020 to April 2024. There were 3,269 likes for the page by the end of April 2024.

The demographics of the followers are listed in Table 2. There were 262 posts on the Facebook page during the period, and we considered 202 posts for analysis. Of these, 132 (65%) posts were related to medical education, 32 (15%) posts were on events, 12 (5%) posts were on posts of interest that are medically related, 11 (5%) posts were on perspectives, eight (3%) posts were on publications and seven (3%) were case reports.

**Table 2:** Demographics

Demographic		Number	Percentage (%)
Gender	Female	1816	54
	Male	1528	45
Age	18-24 years	134	4
	25-34 years	2474	74
	35-44 years	300	9
	45-54 years	200	6
	55-64 years	167	5
	>65	67	2
Geographical region	Sri Lanka	2942	88
	Pakistan	67	2
	Other (Australia, the United Kingdom, India, China, and the USA)	334	10

Considering all the posts, the mean number of reaches per post was 333, and the mean engagement per post was 44. The mean engagement rate of the page was 56%. Of these, the category that had the highest mean reach (1980) was the publications category, followed by case reports, which had a mean reach of 995 per post. The category of posts that had the highest mean engagement was case reports (182), followed by publications (126). The mean engagement rate was highest with the category including posts related to medical education, which was 50%, closely followed by posts related to events at 49% and posts related to perspectives, which had 46% (Table 3).

**Table 3:** Facebook interactions according to the type of post Interactions are defined as a user actively participating in a Facebook post by liking, commenting, sharing, clicking a link, or watching a video.

Post performance metrics	Posts on case report	Posts on medical education	Posts on medical events	Posts on general medical interests	Posts on perspectives	Posts on publications/original articles
Number (n)	7	132	32	12	11	8
Mean reach (n)	995	286	473	140	301	1980
Mean engagement (n)	182	41	33	24	49	126
Mean engagement rate (%)	18.55	50.46	48.95	32.1	45.96	9.48

## Discussion

The Apprentice MSJ and its Facebook platform show a promising approach to enhancing undergraduates’ engagement in academic writing. On Facebook, page “Likes” and “Followers” represent unique Facebook user accounts; therefore, potentially a similar number of real humans have reacted to the page [12]. This can indirectly be used as a measure of popularity [12]. The Apprentice MSJ has garnered almost 3,300 likes, with the majority of followers being university students from Sri Lanka dispersed across the geographic regions of the three main universities in the country, suggesting its relevance to its target audience.

Case reports and research papers were the most popular types of posts. Case reports had a mean reach of 995 and a mean engagement of 182, while research papers had a mean reach of 1,980 and a mean engagement of 126. Overall, the page had a mean engagement rate of 57%. The highest mean engagement rate of 50% was observed in posts related to medical education. Due to the fast-changing nature of the social media milieu, it is challenging to set a precise benchmark for the engagement rate of an academic page to be considered significant. However, these high engagement rates, especially for medical education posts (50%), suggest the platform’s effectiveness in disseminating student research to a wider audience. This is keeping in line with the world data that social media platforms increase the readership of academic medical journals by improving the reach and penetration of the publications [13].

Scientific writing is a skill best planted early in one’s career and developed over time. It is an essential component of the medical profession, and studies have shown that students who publish before graduation are more likely to be scientifically active following graduation [14]. Florek and Dellavalle highlight the importance of training undergraduates to write case reports to provide early career exposure to scientific writing [15]. In our study, case reports and scientific papers were the most popular, indicating a strong interest in scientific writing among the readers. The undergraduate period is a period of transition from layperson to professional, where the students do not fully belong to either category. Having a dedicated platform for their communication is an effective method of enhancing their engagement in academic writing and has gained popularity among local students.

Jha et al. evaluated 225 fourth-year medical students’ views on publishing [16]. Over 90% recognized the importance of starting early, citing lack of mentorship and opportunities to publish as significant barriers. Another study assessing undergraduate participation in research identified a lack of knowledge, attitudes, and limited publishing opportunities as barriers for students from developing countries [17]. Social media platforms like “The Apprentice” offer an alternative to overcome these challenges in undergraduate publishing, especially in developing countries like Sri Lanka. These platforms provide easy access to all students and can be initiated as a student-led activity. Additionally, they can be easily popularized among students, fostering an early interest in writing and the dissemination of ideas across peer groups.

The utilization of social media and mobile devices has become a predominant mode of communication among the younger generation. The rapid popularity of “The Apprentice” among local medical students underscores this trend. Numerous previous studies have highlighted the use of social media in medical education [7,8]. However, the use of Facebook as a platform for semi-structured journals has not been previously reported.

However, there are several key challenges in the progression of social media-based scientific journals. The most critical challenge is ensuring quality assurance and the reliability of data. Although there is a wide variation in quality even among traditional journals, we addressed this issue through staff contributions and adherence to a publishing protocol. Additionally, publishing or posting on social media does not confer the same recognition or validity for authors as traditional publications. Consequently, the ability to attract high-quality publications and the potential to evolve into a top-tier journal are limited in the social media format. Sustaining such efforts often relies heavily on personal interest. Therefore, the authors suggest using this social media platform to complement traditional publishing, rather than compete with it, by serving as a starting point for early-stage writers while guiding them toward more formal publication opportunities.

Nevertheless, there were several limitations in our study. The use of social media metrics like engagement and reach offers limited insight into the students’ skill development. Although they suggest interest among the followers, they do not measure the improvement of students’ critical thinking or writing ability. As writing for the journal and following the journal page are voluntary, these findings might not be generalizable to the entire student population. It is possible that those who engaged in writing already had a background and interest in academic writing. Therefore, our results could be an overestimation of the effect of a social media MSJ among undergraduates.

Future studies should focus on exploring the long-term impact of engaging in social media-based MSJs on students’ writing skills development and career persistence in research [3,4]. Additionally, the replicability of this model should be studied across other developing nations, particularly given the documented disparities in undergraduate research opportunities [5,17]. Comparative studies examining the effectiveness of different social media platforms (e.g., X (formerly Twitter), Instagram, and Facebook) could help optimize student engagement and determine the most effective platform for undergraduate writing development in medical education.

## Conclusions

The demonstrated engagement patterns of this study show that a student-led MSJ utilizing a social media platform can be used as an effective method to engage its target audience of medical undergraduates, with case reports and research articles proving most effective at capturing student attention. The use of a social media platform offers a feasible solution to the limited publishing opportunities available to undergraduates. The use of a student-led editorial panel with the supervision of staff members maintains academic standards of peer review while providing academic writing and publishing opportunities to undergraduates early in their careers. As this social media platform can reach students of multiple universities across the country, it can effectively bridge the gap between informal learning and formal academic publishing, especially in a resource-poor setting.
